# An Improved Forwarding of Diverse Events with Mobile Sinks in Underwater Wireless Sensor Networks

**DOI:** 10.3390/s16111850

**Published:** 2016-11-04

**Authors:** Waseem Raza, Farzana Arshad, Imran Ahmed, Wadood Abdul, Sanaa Ghouzali, Iftikhar Azim Niaz, Nadeem Javaid

**Affiliations:** 1 Department of Telecommunication Engineering, UET Taxila, Taxila 47080, Pakistan; waseemraza844@gmail.com (W.R.); farzana.arshad@uettaxila.edu.pk (F.A.); 2 Institute of Management Sciences (IMS), Peshawar 25000, Pakistan; imran.ahmed@imsciences.edu.pk; 3 Department of Computer Engineering, College of Computer and Information Sciences, King Saud University, Riyadh 11633, Saudi Arabia; aabdulwaheed@ksu.edu.sa; 4 Information Technology Department, College of Computer and Information Sciences, King Saud University, Riyadh 11633, Saudi Arabia; sghouzali@ksu.edu.sa; 5 COMSATS Institute of Information Technology, Islamabad 44000, Pakistan; ianiaz@comsats.edu.pk

**Keywords:** energy efficiency, delay-sensitive routing, forwarding function, holding time, event segregation, network lifetime, throughput

## Abstract

In this paper, a novel routing strategy to cater the energy consumption and delay sensitivity issues in deep underwater wireless sensor networks is proposed. This strategy is named as ESDR: Event Segregation based Delay sensitive Routing. In this strategy sensed events are segregated on the basis of their criticality and, are forwarded to their respective destinations based on forwarding functions. These functions depend on different routing metrics like: Signal Quality Index, Localization free Signal to Noise Ratio, Energy Cost Function and Depth Dependent Function. The problem of incomparable values of previously defined forwarding functions causes uneven delays in forwarding process. Hence forwarding functions are redefined to ensure their comparable values in different depth regions. Packet forwarding strategy is based on the event segregation approach which forwards one third of the generated events (delay sensitive) to surface sinks and two third events (normal events) are forwarded to mobile sinks. Motion of mobile sinks is influenced by the relative distribution of normal nodes. We have also incorporated two different mobility patterns named as; adaptive mobility and uniform mobility for mobile sinks. The later one is implemented for collecting the packets generated by the normal nodes. These improvements ensure optimum holding time, uniform delay and in-time reporting of delay sensitive events. This scheme is compared with the existing ones and outperforms the existing schemes in terms of network lifetime, delay and throughput.

## 1. Introduction

The ocean has been influencing the human race since ancient times. It provides food and sustenance items and plays vital role in weather system, economy and defence of the nations. More than half of the world population live within 150 km from coastline. The conventional exploring mechanism of the oceans involves sending/deploying a sampler in monitoring area of interest especially in the bottom of ocean which collects the required material and then return to surface. There is lack of real time monitoring and retrieving mechanism in case of failure or misconfiguration. Hence these limitations empower Underwater Wireless Sensor Network (UWSN) to become an appropriate candidate for the longstanding and reliable [1] monitoring and data gathering applications. However, UWSN faces lots of challenges due to; the dynamic underwater environment, changing network topology due to water current, and shipping and aquatic life activities. It incurs the substantial cost of deployment, maintenance, and device recovery to cope with unpredictable underwater circumstances. Water absorbs lots of RF (Radio Frequency) energy hence radio communication is not feasible in underwater environments. Hence the communication in underwater is accomplished using acoustic links. Acoustic communication is mainly in low frequency typically less than 30 KHz which limits the available bandwidth and hence lower data rates are observed in underwater communications. The instruments used in underwater devices are also vulnerable to tedious subaquatic challenges, for example, algae and salt accumulation on camera lens, decreasing the effectiveness of sensors and other important devices.

Generally, large number of energy constrained nodes are deployed on different depths to sense the required parameter(s) in underwater environment. These nodes are not aware of their exact position; however, their depth can be found using depth (pressure) sensors. In underwater, nodes communicate with each other through acoustic links. As described earlier, there are some immanent characteristics of acoustic communication like, limited bandwidth (<100 KHz), larger delays, and time varying multi path fading [2] which may cause larger doppler shift. All the deployed nodes also have energy constraint so their efficient energy consumption is of prime importance. It is almost impossible to replace or recharge the batteries of deployed nodes.

The packets generated by each node are transmitted to surface sink using broadcasting and multi-hop communication [3]. In this communication, sending node includes its depth and the routing information in data packet and broadcasts it to all the nodes within its transmission range. However only shallower nodes (i.e., a node with depth lesser than the transmitting node) are allowed to respond to this broadcast. Only those shallower nodes which are farther than the depth threshold become eligible for forwarding. All the eligible nodes holds the packet for their respective holding time which is inversely related to forwarding function. If these packet holding nodes receive the copy of the same packet with-in their holding time they discard the packet and assume that a more eligible node has forwarded the packet. However if holding time is elapsed and no copy is received, node considers itself as the best forwarder and broadcasts that packet. This process is repeated by all nodes till the packet is reached to surface sinks. In this way each time a node with maximum value of forwarding function, gets minimum value of holding time and becomes next forwarder. This communication process involves certain terms which are needed to be explained.

- Depth Threshold: Depth threshold is a global depth parameter to control the number of nodes involved in forwarding process. Greater the values of depth threshold lesser the number of nodes involved in forwarding process. Value of depth threshold is always less than the transmission range.
- Forwarding Functions: Forwarding function is a routing metric which can be dependent on different parameters of nodes, like absolute depth, depth difference, residual energy, transmission range and depth threshold. Different forwarding functions have been defined in literature and included in details in the next sections.
- Holding Time: Holding time is assigned to each node to avoid redundant transmissions and collision. It is the time during which a node holds the packet and avoids any transmission.

Underwater environments are very diverse in nature. A very wide range of living and non living activities take place in the same oceanic area. Generally, this diversity increases as we move deeper into the ocean. There can be many diverse applications in which severity of the sensed value of different nodes may not be the same. The data generated by some nodes may be of more importance than others. This sternness can be of many types like, in terms of delay criticality or in terms of size of data generated. Hence the costly deployment of UWSN can not be dedicated for sensing only a single type of occurring events. These diverse applications require the routing for each type of sensed event. So it is necessary to provide an underwater network solution which considers the diverse events occurrence and appropriate response to that diverse characteristic (different delay criticality associated with different occurring events).

Our contribution in this paper is enlisted in following steps.

- We have considered a diverse environment in which multiple types of events are occurring in the sensing field. These events are named as critical, very critical and normal. Critical and very critical events are delay sensitive in nature so forwarding to surface sink is planned for these types of event using respective forwarding functions in different depth regions. Normal events are sent to the mobile sinks (AUV: Autonomous Underwater Vehicle or CN: Courier Node) patrolling in the network.
- Forwarding functions defined in literature use different parameters in different depth regions. However the values of different forwarding functions must be comparable with each other to ensure the steady flow of packets. Analysis on the range of previously defined forwarding function reveals the incomparable ranges of different forwarding functions. Hence the forwarding functions are redefined to ensure their comparable values in different depth region for the steady flow of packets.
- Mobile sinks are deployed to collect the data from the nodes which has sensed normal events. Hence mobility of these nodes is influenced by the distribution and rate of data generation of normal nodes. We have proposed two different mobility schemes for the mobile sinks.

The rest of the paper is organized as follows: In Section 2 we have provided an extensive literature review about the protocols especially available for underwater sensor networks. The underwater channel model is explained in Section 3. Section 4 consists of problem statement and motivation in which we have identified a problem in previously defined forwarding functions by analyzing their ranges. We also present motivation for event segregation in subsection of this section. Event segregation approach, network initialization and node deployment, improvements in terms of forwarding function and holding time and mobility of courier nodes are explained in the subsections of Section 5. Performance evaluation and result discussion are explained in Section 6. Finally conclusion and future work is presented in Section 7.

## 2. Background and Related Work

As mentioned earlier, underwater network is quite different from the terrestrial networks. The routing protocols for terrestrial networks are broadly divided into two categories: proactive and reactive [4,5]. In proactive protocols-like OLSR (Optimized Link State Routing) [6] and DSDV (Dynamic Destination Sequenced Distance-Vector) [7]-nodes periodically share their routing table information with each other, routes are established evenly irrespective of their requirement. This would cause extra overhead especially in higher lossy underwater links. Whereas in reactive protocols, like AODV (Ad-hoc On Demand Distance Vector) [8], its variants like MRAODV (Multiple Route AODV) [9] and DSR (Dynamic Source Routing) [10]; routes are established on demand initiated from the source. In underwater networks, this route establishing mechanism would be time consuming and causes larger delay due to low propagation speed of acoustic signals. So these protocols are no longer feasible to work in harsh underwater environment [11,12]. There have been many proposed protocols for routing in underwater scenarios [13]. These protocols roughly fall into following two categories. Different aspects of these protocols are given in details.

### 2.1. Localization Based Routing Protocols

In these protocols, position information of each node is calculated with some location finding algorithm. There have been many localization algorithms in literature [14,15,16,17]. A modified version of DSR for underwater environments named as LASR (Location Aware Source Routing) was proposed in [18]. LASR is on demand routing protocol to cope with high latency, high probability of packet loss. It takes into account location and link quality metric during routing process.

Vector Based Forwarding (VBF) was proposed in [19], which aims to increase network lifetime taking into account mobility of nodes. A hypothetical vector, which makes a virtual pipe of radius *R* from source to destination is assumed. Nodes within the range of vector are selected as candidates for forwarding process. EVBF (Efficient-VBF) [20] is an extension and improvement in VBF, in which “desirable-ness” factor *α* and adaptation time $T_{adaptation}$ are utilized in forwarding and selecting most suitable forwarder. This protocol is workable for both sink based queries and source generated queries. Another variant of this protocol is LE-VBF (Lifetime-Extended VBF) [21] in which desirableness is redefined employing position and energy of the deployed node. Routing pipe radius threshold influences on the efficiency of this protocol. VBF also requires localization information and extra equipment to measure Angle of Arrival (AoA) which is an overhead and causes extra energy consumption. Nodes closer to the axis of routing pipe become hot spot and their faster energy depletion causes void regions.

In order to overcome the void area problems faced in VBF, Vector Based Void Avoidance (VBVA) is proposed in [22]. VBVA has capability to detect a void and bypass it on demand, hence it does not need to keep record of topology information. Void avoidance is realized using two mechanisms: vector-shift and back pressure. Void is detected if a node finds no neighbour with “advance” greater than its own “advance”. Vector shift method allows nodes out of routing pipe to forward the packet and hence a shifted vector is formed. If a node cannot find neighbours to shift; it broadcasts a back pressure packet to reroute the packet with better path which has no void. However void detection process is only capable to detect it on the edge of the network and has no solution if void occurs in middle of networks due to unexpected deaths of intermediate nodes. Moreover backward flooding would cause extra over head especially in sparse networks. The performance of these protocols can be improved by identifying critical (i.e., cut-vertices) and non-critical nodes in the network using any of the algorithm such as [23].

Link quality based control and directional flooding protocol is proposed in [24] named as DFR (Directional Flooding based Routing protocol). Link quality is measured in terms of Expected Transmission Count (ETX) and flooding rather than established paths is employed during routing process. Flooding zone is locally formed based on link quality of neighbour nodes. If link quality is poor it involves more nodes in forwarding process otherwise few sufficient nodes are employed. It involves two type of angles: current angle of node *i*; $CA_{i}$ and reference angle of previous sender *k*; $RA_{k}$, in forwarding process. It also provide the mechanism for adaptive change and improvement in $RA_{k}$ by each forwarding node. Geographically controlled flooding zone and directional (toward sink) forwarding based on link quality ensures better packet delivery. However, this protocol requires localization information which is itself a challenge in UWSN. Acquiring link quality information for each node increases control packet broadcasting and causes extra energy consumption. DFR does not have efficient mechanism for redundant packet avoidance.

In Depth Adaptive Routing Protocol (DARP) [25] authors considered the fact that speed of sound varies with depth and proposed shortest delay path for deep sea (about 8000 m) underwater environments. Relative Distance Based Forwarding (RDBF) is proposed in [26]. In RDBF, fitness factor is normalized by transmission range to get the value in range of [0, 1]. Holding time is also dependent on transmission range and speed of sound in acoustic environment. Multi Path Routing (MPT) proposed in [27] ensures balance between energy consumption and end to end delay for time critical underwater applications. Its a cross layer approach which employs: (1) power control on physical layer according to channel status and network conditions; (2) packet combining mechanism on sink for multiple copies of corrupted packets received from different paths. MPT perform well in dense deployment scenarios while suffers void regions in sparse networks.

All the above mentioned localization based protocol requires some collaborative mechanism for to find the location of the deployed nodes. Localization process in harsh underwater environment is itself a research challenge. It also requires some certain number of node to act as Anchor Nodes (AN). Anchor nodes are attached with some support and their fixed location is known. Unknown location nodes communicate with more than one anchor nodes and try to estimate their own location. Error in localization can leads to erroneous communication. Hence its preferred to have localization free routing protocol for UWSN.

### 2.2. Localization Free Routing Protocols

This category consists of protocols which are either completely free of any localization information (based on hop count) or they may require partial localization information DBR (Depth Based Routing) requires only depth). In DBR [28], a receiving node from the set of potential forwarders utilizes its depth and depth threshold (a global parameter) to make the forwarding decision. Forwarding decision is done individually by each receiving node on the basis of receiving time of a certain packet and holding time of that node. Holding time is assigned to each node in such a way that node with lesser depth has less holding time and more probability to be forwarder. Other nodes are prohibited to forward the packet by assigning holding time values more than the optimal forwarder value. DBR performs well to enhance the stability period but when nodes start dying there is no mechanism to rescue and prolong the instability period (time period between the death of first and last node). Throughput of DBR in sparse networks is relatively low, which may require to implement multiple sinks. Additionally, if two nodes lies at same depth then there is no mechanism to suppress the transmission of redundant packets.

Depth Based Multi-hop Routing (DBMR) [29] is a variant of DBR which strives to reduce flooding and redundant packet transmission issues. In DBMR, multi-hop communication is employed during the forwarding process. Weighting Depth and Forwarding Area Division DBR (WDFAD-DBR) is proposed in [30]. Void area occurrence is reduced by selecting next forwarder considering the depth of current node and expected next forwarding node. Weighted sum of depth difference of two hops have role to predict the holding time. There is a mechanism to change the forwarding region adaptively according to node density and channel condition. However neighbour node depth prediction error would lead to increase control overhead.

Another depth based routing protocol is hydraulic pressure based anycast routing called: HydroCast [31]. In HydroCast, firstly an opportunistic routing mechanism is proposed which reduces co-channel interference and ensures maximum delivery. Secondly a simple depth based void area avoidance mechanism is proposed. In HydroCast, void nodes maintain the recovery route to a node who’s depth is lower than its own depth. Then the void node packet is rerouted out of void using greedy forwarding approach. Opportunistic directional flooding is realized in Void Aware Pressure Routing (VAPR) [32]. VAPR operation consists of two parts: enhance beaconing and opportunistic directional forwarding. Additional information such as sender depth, hop count to sonobuoy, sequence number and forwarding direction, is added to each node’s beacon in enhance beaconing phase. VAPR employs local greedy directional forwarding and utilizes a factor called surface reachability as forwarding metric.

A Q-learning based cross layer approach is proposed in [33]. Instead of hop count or depth, Q-learning routing path selection process includes traffic load, latency and queue length. Q-learning in distributed nodes ensures balanced energy consumption. Path Unaware Layered Routing Protocol (PULRP) with uniform deployment of nodes and with non uniform deployment of nodes is proposed in [34]. It consists of two phases, the first phase called the layering phase, in which spherical layers of radius $R_{r}$ with sink as layer 0 are formed and layering number is increased by each node. A node in layer *l* is to find forwarder from layer l−1 on the basis of transmission range and layering radius. The second phase is called communication phase in which potential relay nodes from each layer are selected for packet forwarding. Energy optimized-PULRP [35] is an enhancement in which so called *on the fly* routing is considered to select relay node. E-PULRP incorporate energy in routing decisions, which was not considered in previous versions.

An improvement in DBR named as EEDBR was proposed by A Wahid et al. in [36]. In EEDBR, a sender node maintains depth, residual energy and ID information of its neighbours. Neighbours with smaller depth are selected and their list of IDs is included in data packet sent by sender. Receiving nodes hold the packet for holding time which is based on its residual energy $E_{r}$. This may create problems as two nodes with same residual energy would have same holding time and could transmit at the same time. Moreover, EEDBR involves more number of hops in forwarding process and eventually brings increased delay and enhanced energy consumption. Hop-by-Hop Dynamic Addressing Based ($H^{2}$-DAB) [37] routing protocol is another localization free protocol. In $H^{2}$-DAB, depending on the depth of nodes, different size of addresses are given to different nodes. Deeper nodes have large size address and shallower nodes have less size address. Connectivity based Routing Protocol (CRP) protocol is proposed in [38] in which next forwarder node is directly related to Connectivity Index (CI) of neighbour node. where CI is defined as “number of neighbouring nodes closer to sink from a forwarding node”. Hop count based cross layer approach Channel Aware Routing Protocol (CARP) is proposed in [39]. On the basis of recent history of successful transmissions a particular node is selected a next forwarder. CARP is hop count based protocol with varying power levels.

Another localization free protocol, Adaptive Mobility of Courier nodes in Threshold-optimized DBR (AMCTD), is proposed in [40]. In this protocol, forwarding of packet is improved and based on weight, which is function on depth, residual energy and some priority values assigned to them. An improvement in AMCTD named as improved AMCTD (iAMCTD) is proposed in [41] in which different routing metrics are defined for different depth regions. These metrics include Localization free-SNR (LSNR), Signal Quality Index (SQI) and Energy Cost Function (ECF) and Depth Dependent Function (DDF). Moreover hard and soft threshold based simulation scenario is adopted in which a sensed event’s value greater than hard threshold is reported to sink. Moreover, authors employed courier nodes in [40,41] for data gathering tasks. Here mobility of nodes start when certain fraction of total nodes have died, which is not efficient and full use of deployed resources. Holding time calculation process does not guarantee optimum value of holding time ensuring packet suppression and minimum end to end delay.

## 3. Underwater Channel Model

Underwater wireless channel is severely affected by issues like multi path and fading. Signal to Noise Ratio (SNR) in UWSN is computed using passive sonar equation [42] given below:

(1) SNR=SL−TL−NL+DI

where *SL* is source level of the target or noise generated by the target, TL is transmission loss in aquatic environment, *NL* is noise loss and *DI* is directive index. *DI* is the capability of receiving sensor to direct its antenna to avoid unwanted noise. SNR value must be greater than detection threshold (DT). All the above quantities are measured in dBreμPa. Transmission loss is given in following:

(2) $$TL=10\log\left(d\right)+\alpha \times d\times 10^{-3}$$

Absorption loss α(f) is calculated by the following equation.

(3) $$\alpha_{dB}\left(f\right)=\left\{\begin{array}{cc} \frac{0.11f^{2}}{1+f^{2}}+\frac{44f^{2}}{4100+f}+2.75\times 10^{-4}f^{2}+0.003\; & \mathrm{if}\;f⩾0.4\\ 0.002+0.11(\frac{f}{\left(1+f\right)})+0.011f\; & \mathrm{if}\;f<0.4 \end{array}\right.$$

Thorp attenuation formula [43,44] is used to calculate the totals attenuation *A(l,f)* given in the following formula.

(4) A(l,f)=l×α(f)+k×10log(l)

where *l* is the depth difference *k* is spreading coefficient with value 1.5 for practical spreading. The first term in Equation (4) represents the absorption loss while the second term is about the scattering loss. Passive sonar equation is used to find out Source Level (SL) which is used to find intensity of transmitted signal $I_{T}$ using the following formula.

(5) $$I_{T}=10^{SL/10}\times 0.67\times 10^{-18}$$

Transmission power of source is $P_{T}\left(d\right)$ calculated by the following equation.

(6) $$P_{T}\left(d\right)=2\pi \times 1m\times H\times I_{T}$$

Energy consumed in transmitting the *k* bits of packet is given as:

(7) $$E_{Tx}\left(k,d\right)=2P_{T}\left(d\right)\times T_{Tx}$$

where $T_{Tx}$ is the transmission time in seconds. Speed of the acoustic signal is calculated by the following equation.

(8) $$v=1499.05+45.7t-5.21t^{2}+0.23t^{3}+\left(1.333-0.126t+0.009t^{2}\right)\left(S-35\right)+16.3z+0.18z^{2}$$

Average end to end delay consists of two type of delays, calculated and explain in the following equations.

- Transmission delay: Transmission delay to a packet of length *l* caused by a node *n* is given by following formula.
  (9) $$D_{t}^{n}=\frac{l}{r}$$
  where *r* is data rate with value 250,000 bps.
- Propagation delay: Propagation delay of an acoustic signal moving with velocity *v* between a sender *i* and receiver *j* separated by distance $s^{i,j}$ is given by:
  (10) $$D_{p}^{i,j}=\frac{s^{i,j}}{v}$$
  The speed of acoustic signal v= 1500 ms${}^{-1}$ which is calculated using Equation (8). Sum of both type of delay constitutes to one hop delay.
  (11) $$D_{n}^{i,j}=D_{t}^{n}+D_{p}^{i,j}$$

The sum of all hops delay equals to the total delay in a packet transmission from a certain node *n* to sink. It is also termed as end to end delay.

(12) $$D_{e2e}^{n}=\sum_{h=1}^{h_{max}}D_{h}$$

The mean of all end to end delays gives average end to end delay.

(13) $$\bar{D_{e2e}}=\frac{\sum_{n=1}^{n_{max}}D_{e2e}^{n}}{n_{max}}$$

## 4. Problem Statement and Motivation

This section is summarized in following points.

- Initially, we present a comprehensive analysis on the ranges of forwarding functions defined in previous protocols. We have identified problems in those forwarding functions and redefined them in next section. These redefined forwarding functions causes much better and systematic forwarding.
- In second part of this section we explain broadcasting overhead issue in deep sea acoustic networks and motivation for event segregation approach.

### 4.1. Forwarding Functions Analysis

Some important aspects of communication in underwater are neighbor selection, forwarding process (function) and holding time calculation. In order to predict the forwarding behaviour in network and its impact on holding time of nodes, it is convenient to have knowledge of domain and range of the forwarding functions. We have done comprehensive analysis on the respective forwarding functions defined in [40,41]. The upper and lower bounds on the affecting metrics are given in Table 1. If the deepest layer of network is considered as reference level, $h_{i}$ = $D_{max}-D_{i}$ gives the information about the height of node.

DBR and other depth based protocols select neighbors on the base of depth difference Δd between the depth of previous ($d_{p}$) and current node ($d_{c}$). Holding time in DBR is also function of depth. Depth of broadcasting nodes can have range from; transmission range $R_{T}$ to maximum depth $D_{max}$, which directly affects the holding time calculations. So this fails to explain the upper and lower bounds on possible holding time values while considering the delay and packet suppression tradeoff.

EEDBR selects next forwarders on the basis of depth and residual energy. A node during its lifetime can have energy between 0 and $E_{o}$. Hence the range of forwarding function in EEDBR is between 0 to $E_{o}$. Weight based forwarding function in AMCTD is dependent on both remaining energy and depth difference and is given in following equations:

(14a) $$W_{1}=\frac{p_{v}\times E_{r}}{D_{max}-D_{i}}$$

where $E_{r}$ and $D_{i}$ are the residual energy and absolute depth of ith node respectively, $p_{v}$ is priority value which is a system parameter. $D_{max}$ is the maximum depth of underwater network. Weight calculation in AMCTD is updated and shifted more on remaining energy after 2% death of total nodes using following equation.

(14b) $$W_{2}=\frac{p_{v}\times D_{i}}{E_{r}}$$

In order to prolong the lifetime in sparse conditions, weight calculation is updated after 80% death of total nodes by Equation (14c).

(14c) $$W_{3}=\frac{E_{r}}{p_{v}\times D_{i}}$$

For a unit value of $p_{v}$ all the forwarding function $W_{i}$ calculated for AMCTD using Equation (14) has range in interval [0,∞). Holding time values depending on this forwarding function would vary abruptly and would not meet the optimum delay and reduced redundant transmission requirements. In AMCTD depth threshold $d_{th}$ is also updated after certain percentage of deaths, but weights are independent of $d_{th}$ and transmission range $R_{T}$ of nodes. Anyhow if there comes a lower bound on $E_{r}$ defined as $min(E_{r}\geq 1)$ and $D_{i}$ defined as $min(D_{i}\geq 1)$ it causes a significant change on the upper bound of all the respective forwarding function $W_{i}$ as in Table 2. Since forwarding function of AMCTD are independent of depth threshold so maximum values are same regardless of $d_{th}$. Dimensional analysis of each forwarding function describes that $W_{1}$ and $W_{3}$ are dimensionally equivalent (=Newton) while $W_{3}$ is dimensionally reciprocal of $W_{1}$ and $W_{3}$.

Forwarding function in iAMCTD is defined differently for different depth regions. SQI based forwarding function is defined in Equation (15a).

(15a) $$SQI=\frac{LSNR\times E_{r}}{l_{i}}$$

In this equation $l_{i}$ is depth difference between two successive nodes. LSNR is Localization free Signal to Noise Ratio defined in [41] and given by the following equation.

(15b) $$LSNR=\frac{P_{t}}{A(l,f)\times N(f)}$$

where $P_{t}$ is transmitted power and (A()×N()) is the attenuation noise product factor, which is product of path loss and ambient noise. A(l,f) is the sum of absorption coefficient (Abc) and scattering coefficient (Sc) whose minimum and maximum values for different depth thresholds are given in Table 1.

SQI based forwarding decisions are made if depth of the node is less than $D_{1}$ which is top shallow region of underwater environment. This region involves more shipping activities. We have considered the statistical fact that variance is the measure of randomness in a set of observations. Careful analysis of SQI reveals that the range of output from this function is in the order of $10^{5}$ as shown in Table 1. Such values must not be used directly as forwarding function as holding time calculated from these values would be very much incomparable, having high value of variance. So these values are needed to be made comparable before being used as forwarding function and holding time. This is explained as follows:

If *f* = 30 kHz and depth difference *l* has range in the interval [$d_{th}=60,R=100$] absorption coefficient has range [0.5969 dB, 0.9949 dB]. A(l,f) eventually comes out with in the range of [533.2,1245.4]. For *f* = 30 kHz noise N(f) comes out to be $1.12\times 10^{-5}$ using this, Attenuation Noise product range is calculated and finally LSNR is in range of [$4.52\times 10^{6},\;1.06\times 10^{7}$] making SQI in range of [$0,\;1.01\times 10^{5}$]. This analysis is for $d_{th}=60$ m but when $d_{th}$ is modified to 40 and 20 value of forwarding function for different nodes would be with larger values of variance. Direct use of values in this range is impractical and is needed to made comparable with other forwarding functions. Hence, in our solution linearized version of SQI is proposed to be used as forwarding function.

ECF based forwarding function is utilized when depth of node is between $D_{1}=150$ and $D_{2}=350$. ECF is defined as:

(15c) $$ECF=\frac{p_{v}\times E_{r}}{D_{i}}$$

where $D_{i}$ can be between $D_{1}$ and $D_{2}$. Value of $E_{r}$ is between 0 and 70 J and this results ECF values between [0, 70] which is very different from the range in SQI based forwarding function. ECF is also modified to include transmission range and depth threshold. If node is in deepest region of networks with depth greater than $D_{2}$, DDF based forwarding function is employed using following equation.

(15d) $$DDF=\frac{SQI\times l_{i}}{D_{i}}$$

Range of DDF is also given in Table 2. For a certain value of depth threshold $d_{th}$ maximum and minimum values of DDF are roughly one tenth of the corresponding SQI function.

Holding time is the time for a certain node during which it avoids any transmission and waits for the more appropriate forwarder to transmit the packet. Its value for each node is related to forwarding function of the underlying scheme. Difference of values of holding times for two successive nodes should be sufficiently large enough to avoid any collision. On the other hand very large value of holding time causes delay during transmission process. This delay may be negligible on a single node but in multi hop communication accumulated sum of all delays eventually causes larger considerable delay. So an optimum value of holding time is required to ensure redundant packet suppression and minimum delay. Holding time calculated for AMCTD and iAMCTD is given in following equation.

(16) $$H_{T}=\left(1-F_{F}\right)\times H_{tmax}$$

Holding time calculation depends on two factors $H_{tmax}$ and forwarding function $F_{F}$. There is no appreciable explanation of $H_{tmax}$ which is termed as system parameter in previous literature. Whereas values for different forwarding functions can have range very much different from each other and these values are not even comparable with each other. Holding time calculated with these forwarding functions has very abrupt fluctuations for different depth nodes which causes increased delay in some regions and redundant transmission and collision in other depth regions. So there is need to have a relation providing comparable values of holding times. At least one must be able to predict the range of values to be assigned as holding time.

### 4.2. Motivation for Event Segregation Approach

Much of contemporary work in underwater sensor networks strives to have an effective routing mechanism ensuring decreased energy consumption and increased network lifetime. Each node strives to find a set of best possible nodes for the successful delivery of packet to surface sink. This situation (forwarding all the data to surface sink) would be appropriate if data generating nodes are only a fraction of all the deployed nodes. However in some applications in which all the deployed nodes are equally probable to sense and generate data packets, this approach causes increased broadcasting overhead. Each node has its own data plus it may has to forward the packets of other deeper nodes. This issue becomes prominent in deep underwater environment which involves comparatively greater number of hops in forwarding process. In this forwarding scenario node would be wasting its resources by causing congestion and increasing probability of collision of packets. Hence it is no more a wise option to broadcast all the generated data to surface sink(s). There is a need to decrease the broadcasting load on intermediate nodes to ensure optimum energy consumption. This becomes one of many motivation for the formulation of event segregation approach.

One possible solution to decrease broadcasting load in some recent work [41] is to deploy certain number of underwater mobile sinks (AUV or CN) which can move to the appropriate location for data collecting jobs. But it requires proper tour planning and coordination between nodes and sinks. Nodes may have to extra memory to hold the data for some time till the sink arrival. It would also cause increased delay and more susceptibility of congestion, collision and increased packet loss. It also requires a decisive parameter for a node so that it can be decided whether to forward to mobile sink or broadcast to shallower nodes. This broadcasting choice needs to be dependent on some decisive criteria. The event segregation approach provides an important criteria for this decision making step.

Moreover conventional underwater protocols are generally planned for single application in which all sensing nodes are assigned the task of sensing a particular type of event and reporting to the floating sink. Occurring events have same occurrence probability in sensing field and require similar delay sensitivity for all nodes. These protocols provide no solution for diversified applications in which multiple events, with different tolerable delay requirements, can occur with different probability of occurrence. Moreover, generally UWSN are considered delay tolerant in which major constraint is energy consumption while it is assumed that there is relaxation in terms of delay. This is over simplified assumption as some events although with very less probability of occurrence may require to be reported much earlier than normal events. Event segregation approach considers this delay sensitivity associated with every node and plans more efficient approach for packet delivery.

## 5. ESDR: Event Segregation Based Delay Sensitive Routing

In this section our proposed protocol Event Segregation based Delay sensitive Routing is introduced and salient features of ESDR are explained. Directed Graph (digraph) theory is employed to serve as model for network topology in ESDR. Deployed nodes map to vertices and links between nodes represent edges of digraph. In digraph G=(N,A) vertices N are connected via edges or arcs A. Where N is the set of all deployed nodes. We denote the set of event sensing or data generating nodes by E, the set of broadcasting nodes by B and the set of courier nodes by C.

There are some applications where only a fraction of all the deployed nodes are the data generating nodes, i.e., the members of E while the remaining nodes are the members of set B. While both the set E and B are disjoint and subset of set N. There are some other (regular data gathering) applications in which all nodes are equally responsible for data sensing and forwarding tasks. In such cases the set E is equal to set B and both sets are subset of set N.

We have considered the second case in which all nodes can be both the data sensing and forwarding nodes. The upcoming subsections provides the details of the phases involved in the protocol operations. These are summarized in the following points.

- In first subsection network initialization and node deployment is explained.
- Motivation for event segregation and its positive impacts on network are explained in this subsection.
- Improvements in terms of forwarding function calculation process and holding time calculation process is explained in subsequent subsections.
- Mobility of mobile sink is also impacted by event segregation approach. Mobility of deployed courier nodes in ESDR is explained in this subsection.

### 5.1. Network Initialization and Node Deployment

ESDR considers deep underwater environment which comprises of *l* m × *b* m × *D*${}_{max}$ m. Initially N nodes having same initial energy $E_{o}$ are randomly deployed. Each node is equipped with required sensing capabilities and a communication module. Each node is aware of its depth which is calculated using pressure sensors. We have considered the following assumption about the deployed nodes and network.

It is assumed that the horizontal mobility of nodes is negligible while the vertical motion of nodes may change their depth. However, since it is a localization free scheme depending on the instantaneous depth, the change in depth is already accounted in the protocol operation. Hence the impact of mobility of water on nodes position is not taken into consideration. It is also assumed that each sensing node on the basis of value of sensed attribute can determine its type and delay criticality associated with it. One way to realize this assumption is by predefining some threshold levels of the being sensed parameter and then conditioning their type by the threshold values. After sensing the event a node determines its types by comparing the sensed value with the thresholds.

**Figure 1:**
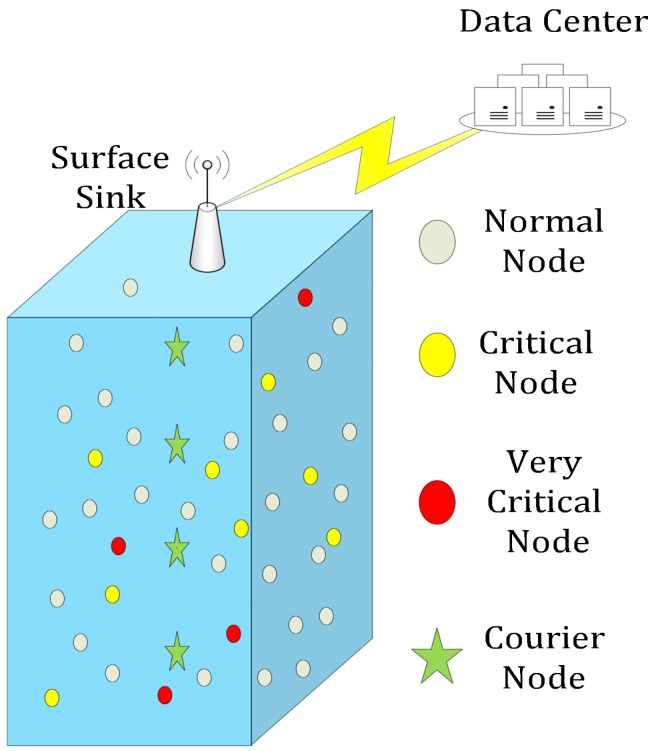
Node deployment.

Four courier nodes are also placed at different depths as shown in Figure 1. Courier nodes are deployed for data collection and have the capability of vertical motion.

**Figure 2:**
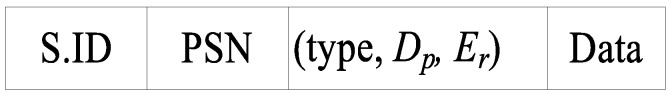
Packet format.

Packet format for ESDR is shown in Figure 2. S.ID is sender ID, PSN is packet sequence number, depth of previous sender is kept in $D_{p}$, $E_{r}$ is the remaining energy of previous node. One important field is packet type which can be very critical, critical or ordinary.

### 5.2. Statistical Event Generation and Segregation

One of the unique aspects of ESDR is event segregation approach as it considers delay sensitivity associated with different types of events and assures efficient delivery to appropriate destination. We have devised a statistical model which considers different events occurring in sensing field. It is assumed that sensing node is able to determine the type of a particular sensed event and delay criticality associated with that event. In order to simplify the analysis occurring events are divided into three types: very critical, critical and normal/ordinary with the probability of occurrence $p_{vc}$, $p_{cr}$ and $p_{no}$ respectively. Nodes with critical and very critical events are collectively termed as delay sensitive nodes and their data is termed as delay sensitive data. The nodes of each type of events are members of the sets defined as $E_{vc}$, $E_{cr}$ and $E_{no}$. Cardinality of these sets of node is given as:

(17a) $$|E_{vc}|=\lfloor p_{vc}\times \left|E|\right\rceil$$

(17b) $$|E_{cr}|=\lfloor p_{cr}\times \left|E|\right\rceil$$

(17c) $$|E_{no}|=\lfloor p_{no}\times \left|E|\right\rceil$$

where sign ⌊⌉ represent the round off decimal value to nearest integer. The probability values are set as $p_{vc}=0.111$, $p_{cr}=0.222$ and $p_{no}=0.666$ used in simulation section, which describe the number of nodes in each type of set. These values are assigned on the base of the assumption that more critical events are less likely to occur and vice versa. The values assigned to these probabilities ensure that; (i) the proportion of each type of nodes remain same throughout the network operation and (ii) the sum of all types of nodes is equal to the total number of data sensing nodes |E| as given by the following equation.

(17d) $$|E_{vc}\left|+\right|E_{cr}\left|+\right|E_{no}\left|=|E\right|$$

Considering the above mentioned event distribution and the probability values, it is ensured that a specific node of its lifetime *r* units (rounds or seconds), ⌊66.6×r⌉ times it would sense ordinary event, ⌊22.2×r⌉ times it would sense critical event and ⌊11.1×r⌉ times it would be very critical event. This probability also ensures that at any time, ⌊66.6×|E|⌉ out of |E| event sensing nodes would be ordinary nodes, ⌊33.3×|E|⌉ nodes would be critical and ⌊11.1×|E|⌉ nodes would sense very critical event. However in this model nodes are selected randomly as critical, very critical and normal but it is ensured that the length of each set of node is not changed.

### 5.3. Packets Forwarding Strategy

In our proposed protocol, it is envisioned to formulate broadcasting mechanism for all types of nodes considering the delay sensitivity requirements of critical and very critical nodes. In our proposed protocol, we have planned the task dividing approach in which packets are forwarded either to the sink (floating on surface) or to CN based on their type and criticality requirements. Sojourn tour of CN can cause larger value of delay as data collected by CN is delivered to data center after its complete round trip. Hence it is ruled out to forward delay sensitive packets to CN. On the other hand, it is also inadequate to forward all the generated packets to surface sink(s) especially in deep underwater environments or in regular data gathering applications. That is why it is planned that delay sensitive nodes will forward the packets to surface sink using broadcasting and multi hop communication approach. Improved forwarding functions for critical and very critical packets are explained in upcoming sections. Forwarding function involves priority values $p_{v}$ whose value is increased to distinguish the forwarding of very critical packets from the critical packets. Normal nodes try to forward or broadcast their respective packets to CN. Hence CN mobility is planned and influenced as per normal nodes distribution and data generation. Broadcasting of normal node’s packets to CN can also involve one hop communication. If a normal node has packet to forward and it is not in the range of CN then it would check if any of its neighbours in the range of CN such that the packet is sent to that neighbor which relays the packet to the nearest CN. In order to accomplish this, normal nodes share their neighbor list with each other. Disjoint neighbors are updated by each node. Nodes with depth less than transmission range directly send their data to surface sink irrespective of their type.

### 5.4. Improvements in Terms of Forwarding Function

The forwarding function is the criteria for a node to decide whether to forward the delay sensitive packets or not. Forwarding decisions are made by each node irrespective of its own type. This means a normal node; which forwards its own packets to mobile sinks and do not broadcast to the surface sink, receives a delay sensitive packet it would be eligible for forwarding and forwards the packet if it comes out to be the best forwarding node. Likewise with iAMCTD, the forwarding function in ESDR is defined differently for different depth nodes. The SQI based forwarding function is redefined to ensure the comparable values of the forwarding function.

(18) $$SQI^{\prime }=\frac{\log_{10}\left(LSNR\right)\times E_{r}\times R_{T}}{l_{i}}$$

The SQI based forwarding function range is always in interval [0, 491.91]. The upper limit is far less than defined in previous schemes and is more comparable to the range of ECF and DDF based forwarding function. ECF based forwarding function $F_{F}$ is directly proportional to $E_{r}$ and inversely proportional to depth difference previous and current node depths $D_{p}-D_{c}$ is defined below:

(19) $$ECF^{\prime }=\left[p_{v}\times \frac{R_{T}}{D_{p}-D_{c}}\right]E_{r}$$

where $R_{T}$ is transmission range $E_{r}$ is remaining energy of a certain node. Forwarding function is directly dependent on remaining energy weighted by a factor given in square brackets whose range is described as follows: Neighbour selection process and depth threshold ensure that $D_{th}<\left(D_{p}-D_{c}\right)<R_{T}$. Initially $D_{th}$ is set to 60 m and is subsequently changed to 40 m and 20 m after certain percentage of deaths, and $R_{T}$ is constant =100. So for above mentioned values factor in square brackets in Equation (19) has range, from 1 to 1.66 for $d_{th}=60$, from 1 to 2.5 for $d_{th}=40$ and from 1 to 5 for $d_{th}=60$. This eventually gives value of forwarding function always between 0 to $5\times E_{o}$ which is comparable with the range of ${SQI}^{\prime }$. DDF is also modified taking newly defined and linearized version of SQI.

(20) $$DDF^{\prime }=\frac{SQI^{\prime }\times l_{i}}{D_{i}}$$

SQI based forwarding function is employed when depth of nodes is less than $D_{1}^{\prime }=250$ m, ECF based forwarding decision is used if node is in between $D_{1}^{\prime }=250$ m and $D_{2}^{\prime }=750$ m. Nodes with depth more than $D_{2}^{\prime }$ forward the packet using DDF based forwarding function.

### 5.5. Improvements in Terms of Holding Time

Holding time is given to nodes with lesser values of respective forwarding function. Holding time calculation is defined by the formula given in equation.

(21a) $$H_{T}^{\prime }=\left(1-F_{F}\right)\times H_{T}$$

$H_{T}$ is the maximum value of holding time. It is defined in following equation as the ratio of consumed energy per unit power.

(21b) $$H_{T}=\frac{E_{o}-Er}{P_{T}}$$

So if a certain node has less value of remaining energy it would have higher value of holding time and refrain to forward the packet.

### 5.6. Mobility of Mobile Sinks

Courier nodes and AUV are employed for data gathering applications hence they are very crucial for underwater networks. Courier nodes can move vertically up and down and there is no facility of horizontal movement while AUV can be moved to any desired place with proper tour planning. However, there is no energy constraint on both of these devices. The mobility of courier nodes has not been exploited to its full potential in previous work. In most of the previous schemes, mobility of these nodes starts after certain percentage of death. Attaining the global knowledge of deaths and updating that to CNs itself has communication overhead. Additionally employing a resource in deep water and waiting for some certain level of calamity is not a wise option. Mobility of these nodes is needed to be exploited as per network requirements. So it is necessary to plan a better mobility pattern of CN to get full use of it. We have reconsidered the deployment and mobility of CNs. Moreover event segregation dictates and selects limited number of nodes to forward to CNs. Hence mobility of courier node is influenced by density of normal nodes and their rate of packet generation. In our proposed environment horizontal movement of CN is not required while vertical movement of each courier node is explained in two different ways. It is worthy to note that the mechanical aspect of the motion of mobile sink is beyond the scope of this work. Hence it is ignored that how and at what cost; these mobile devices move and what is the impact of underwater environment on this motion.

#### 5.6.1. Synchronized and Uniform Mobility of AUV

**Figure 3:**
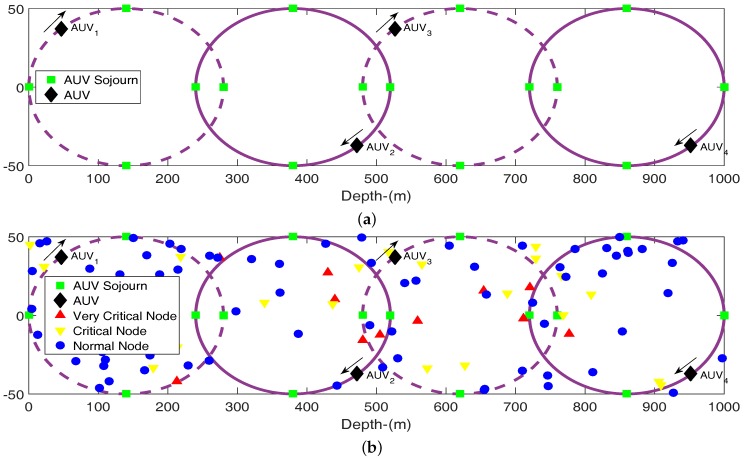
Synchronized mobility of AUV. (**a**) Uniform mobility of AUV; (**b**) Uniform mobility of AUV with node deployed nodes.

In this type of mobility four courier nodes deployed on four different depths move in elliptical path. The centers of the four ellipse are given in Table 3. The motion is uniform in the sense that speed of each AUV remains same in its sojourn tour on elliptical path. By the term synchronized mean that phase relation between any two AUVs remains same throughout the tour as shown in Figure 3. In this figure two ellipses are shown by solid line and two by dashed line. The both AUVs of solid line ellipses move in same direction and remain in phase with each other, while both the dashed line AUVs are in phase, move in the same direction but opposite to the previous pair of AUV. The green dots in these figures represent the stopping points of AUVs.

Elliptical path of courier node *c* in *yz* plane is defined in following equation.

(22a) $$\frac{{\left(z-k_{c}\right)}^{2}}{a^{2}}+\frac{{\left(y-h_{c}\right)}^{2}}{b^{2}}=1$$

Elliptical path is planned in such a way that two ellipses have some overlapping region as depicted in Figure 3a. This allows better coverage to whole region and also facilitate to respective lower AUV to send its data to upper AUV. If the length of overlapping region on *z* axis is *x*, semi major axis length *a* is related with *x* by following equation.

(22b) $$a=\frac{3}{8}x+125$$

**Figure 4:**
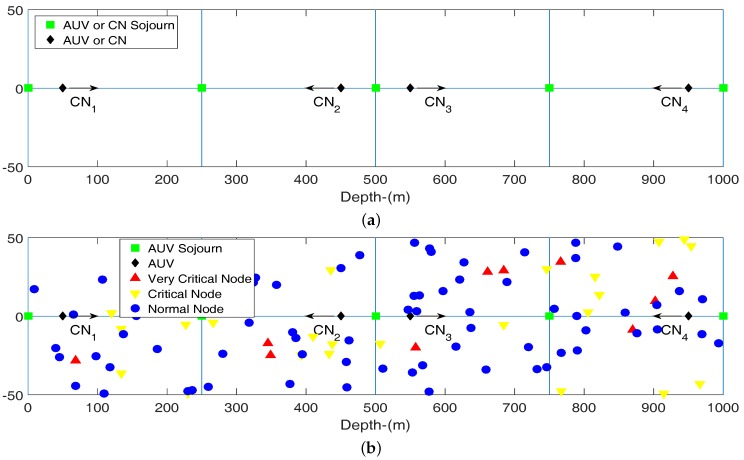
Adaptive mobility of courier nodes. (**a**) Adaptive mobility of courier nodes; (**b**) Adaptive mobility of courier nodes with deployed nodes.

This relation reveals that for x=0, *a* becomes equal to 125 making length of major axis of the each ellipse to 2a=250. *x* is subsequently changed to 20 and 40 making a=133 and 140. The resulting ellipses are shown in Figure 4. In this type of mobility vertex length a=140 and co-vertex length b=50 are used. In order to calculate time period of a node it is necessary to have knowledge of perimeter (circumference) of an ellipse. There is no straightforward formula to calculate this and following infinite series is utilized.

(22c) p=π(a+b)∑n=1∞0.5n2hn

The parameter h is defined as follows:

(22d) $$h=\frac{{\left(a-b\right)}^{2}}{{\left(a+b\right)}^{2}}$$

We have employed the following formula by Ramanejan [45] to calculate the perimeter of the each elliptical.

(23) $$p\approx \pi [3\left(a+b\right)-\sqrt{\left(3a+b)(a+3b\right)}]$$

Time period of each nodes is dependent on uniform tangential speed of courier node during its sojourn tour. In synchronized and uniform mobility pattern speed of a node *i* is $v_{i}$ making time period:

(24) $$T_{i}=\frac{p}{v_{i}}$$

Mobility of CN is envisioned in such a way that $CN_{1}$ is in phase with $CN_{3}$ and $CN_{2}$ is in phase with $CN_{4}$. Initially $CN_{1}$ and $CN_{3}$ are at bottom end of their respective elliptical paths and $CN_{2}$ and $CN_{4}$ are at the top end of their corresponding elliptical paths. Every node moves in clockwise direction and reaches to next destination after time $T_{i}/4$. In this way a node in its one revolution has four points to stop each after phase angle of θ/2. Their motion is synchronized in such a way that when $CN_{1}$ is about the top. Each node moves upward to their predefined destination, transfer data to next CN, and move back to initial stage. This motion pattern is synchronized in such a way that when a certain courier node is about to reach its destination next forwarder has just started its tour, first node find next CN to take its data to next CN.

#### 5.6.2. Adaptive Mobility of Courier Nodes

In this mobility pattern, nodes may accelerate or decelerate their speed on the basis of node density and data generated by the upcoming nodes. In this motion, nodes move up and down on a vertical line and their speed is influenced by node density and traffic generation rate of normal nodes. Courier nodes move in pairs as mobility of $CN_{1}$ and $CN_{3}$ is in one direction while $CN_{2}$ and $CN_{4}$ move towards other direction. This type of motion pattern ensures that these nodes must overlap and communicate with each other at the overlapping area. In order to illustrate the motion of a courier node *c* consider it is at position $y_{c}$; its new position $y_{t}$ after time *t* can be determined by following equation.

(25) $$\frac{dy_{c}^{2}}{dt^{2}}+\frac{1}{t}\frac{dy_{c}}{dt}+y_{c}=\frac{1}{t^{2}}\times y_{t}$$

In this equation left term (second degree differential term) corresponds the acceleration of courier node *c*. If this value is set to zero motion becomes uniform. For adaptive mobility of the nodes, this factor is dependent on the node density in the upcoming coverage region of courier node *c*. Upcoming coverage regions is double of the transmission range of courier node. This process is explained as follows: Initially a courier node *c* increases its transmission range to double of the present value and broadcast its arrival to its future region. Receiving nodes acknowledge with their depth information, rate of data generation and elapsed time since their previous transfer of data to any courier node. The courier node calculates the mean of the depth information of only those nodes which are currently not in its range. This provides that spatial information to estimate its next possible location in its sojourn tour. It also utilizes elapsed time data to calculate the allowed time to reach the estimated location. Using the depth difference between current depth and calculated depth; and remaining time node courier node *c* decides new velocity to reach at next location. Courier nodes keep the record of this location and utilizes it in its next sojourn tour. Hence location estimation of courier nodes is a gradually improving process.

(26) $$y_{t}=\frac{1}{N_{d}}\sum_{1}^{N_{d}}D_{i}$$

where $N_{d}$ represents total number of data generating nodes farther than current transmission range of courier node. Node utilizes elapsed time and average traffic generation of all nodes $N_{d}$ to determine remaining time $t_{r}$ to reach new location. This remaining time $t_{r}$ along with new location distance $y_{t}$ are utilized to estimate the optimum velocity for next part of tour.

(27) $$v_{c}=\frac{y_{t}}{t_{r}}$$

Variations of $v_{c}$ during its sojourn tour make sure the adaptive and accelerated mobility of courier nodes for reaching at optimum new location with in the appropriate time.

## 6. Performance Evaluation

We have considered the deep underwater environment of volume 100 m × 100 m × 1000 m in which 224 nodes are randomly deployed. Analytical calculations are programmed in MATLAB using channel model equations. Generally in UWSN applications can be divided into two major groups on the basis of traffic generation. In some applications only a fraction of all deployed nodes are event sensing nodes which can generate data packets. The rest of the nodes are used to forward or broadcast that data. In other group all the deployed nodes can sense and communicate equally. The number of nodes in former group can be fixed and preselected based on their depth, or can be varying and adaptively selected based on some threshold value of sensed attribute. We have chosen the second group in our simulations in which all the deployed 224 nodes are data generating nodes and also act as forwarder to the received data packets. In simulation scenario each node is allowed to generate exactly one packet in one round.

Simulation setup for random event generation of ESDR can be with many different possibilities. This number can be fixed or varying (as in present case we have chosen fixed number of node in each type). For example in this scenario nodes are selected randomly as critical, very critical and normal. This election is repeated after 100 rounds; however, it is ensured that total number of nodes in each type remains constant. So every time, a network ratio of very critical, critical and normal nodes is constant and it remains uniform for the whole lifetime of network. One can argue about varying numbers of delay sensitive nodes because who knows how many events can be delay sensitive. However, in order to simplify the analysis and simulation setup, so far a constant ratio of delay critical nodes is used.

We have compared the performance of ESDR with DBR, EEDBR and iAMCTD. In order to ensure fair comparison all the comparing schemes are implemented in same sensing volume. Necessary parameters with their values are given in Table 4.

### 6.1. Performance Evaluation

In order to compare the performance of underlaying schemes following evaluation metrics are considered.

- Stability period: Time from the start of network operation till the death of first nodes is termed as stability period, whereas time from the death of first node till the death of last node is termed as instability period.
- Halflife: Halflife of network is defined as the time till the death of half nodes.
- Lifetime: Lifetime of network is the time till the death of all nodes.
- Average end to end delay: Average end to end delay is mean of accumulated sum of propagation and transmission delays of all nodes.
- Throughput: Throughput is considered in terms of following two aspects:
  1. Packet sent to and received at sink: Number of packets sent to sink are dependent on type of application. In event driven or threshold sensitive applications some selected nodes are packet generator nodes, while in other type all the nodes have equal probability to generate the packet after some regular interval and sent to sink. Number of packets successively received at sink is less than the number of packets sent to sink.
  2. PDR—Packet Delivery Ratio: PDR is the ratio of number of packets received at sink to number of packets sent to sink. In ideal case where no packet is dropped PDR is equal 1, otherwise its value is less than 1 and greater than 0.
- Transmission loss: Transmission loss is the average decrease in sound energy/intensity level as it propagates through water.

Each metric is explained in details in the following subsections.

#### 6.1.1. Network Lifetime

**Figure 5:**
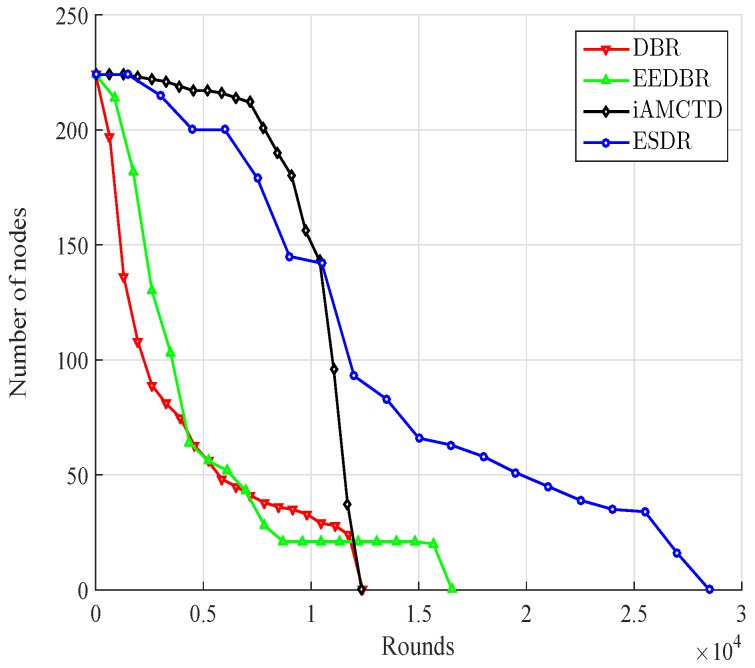
Network lifetime.

Network lifetime is very crucial evaluation metric for the progress of any UWSN protocol. It is always required to prolong the lifetime with some efficient routing decisions considering the affecting parameters. Network lifetime is dependent on the energy consumption of deployed nodes which is calculated using Equation (7). Network lifetime of the underlying schemes is given in Figure 5. Stability period of ESDR is greater than iAMCTD after that iAMCTD shows somewhat stable behavior and there occurs rapid deaths in ESDR. This is due the difference of courier node mobility strategy between iAMCTD and ESDR. In iAMCTD courier nodes mobility is associated with the certain percentage of deaths. Hence courier nodes come into action as nodes have depleted a big portion of their energy and about to die out. Courier nodes data collection reduces large distance transmissions and bring somewhat stable behaviour in network. Anyhow this rescue mechanism could not prolong lifetime to very much extent. Halflife of both schemes is almost similar but after that iAMCTD nodes die out very rapidly whereas ESDR prolongs its lifetime to appreciable extent. Motion of courier nodes in ESDR is influenced by relative distribution and data generation of normal nodes. This motion is cyclic in nature in which, in each new cycle courier node utilizes record of its previous sojourn stay and estimates the better new position. This gradual improving mobility mechanism results into the deterrent behaviour of network. This type of mobility strategy may not stop the deaths but the lifetime of more important nodes (with higher rate of data generation) is prolonged while the death of lesser important nodes is tolerated for the overall benefit of network. iAMCTD nodes die out very early due to lack of network healing mechanism. Over all comparison proves that ESDR outperforms iAMCTD, EEDBR and DBR by considerable margin.

The lifetime of all the comparing schemes are different from each other. Hence lifetime cannot serve as a fair independent quantity for comparison of remaining evaluation metrics. So in order to have a common independent quantity we have taken certain but equal number of ascending samples of each respective metric, from the timeline of all the comparing protocol. Then these taken samples of the respective metric are plotted and compared. So samples in timeline of following figures actually refers to the equally spaced and same number of samples of each evaluation metric taken in from each protocol.

#### 6.1.2. Average End to End Delay

**Figure 6:**
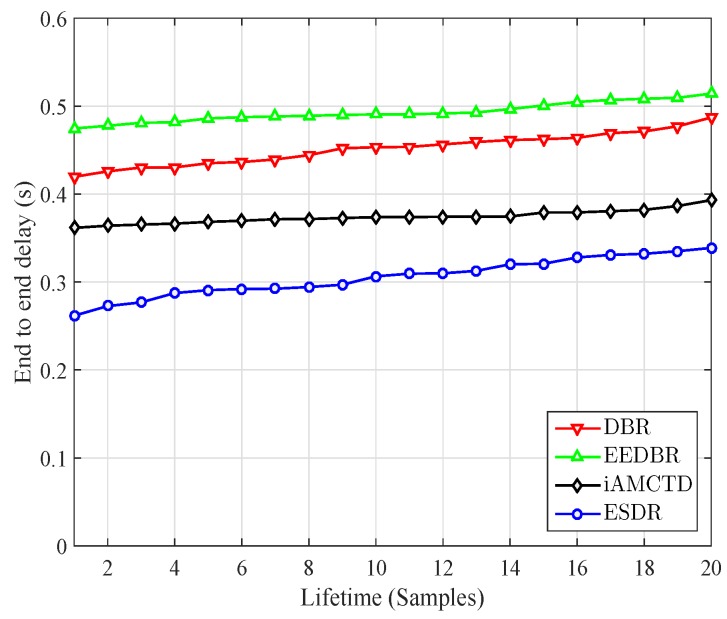
Average end to end delay: Comparison.

**Figure 7:**
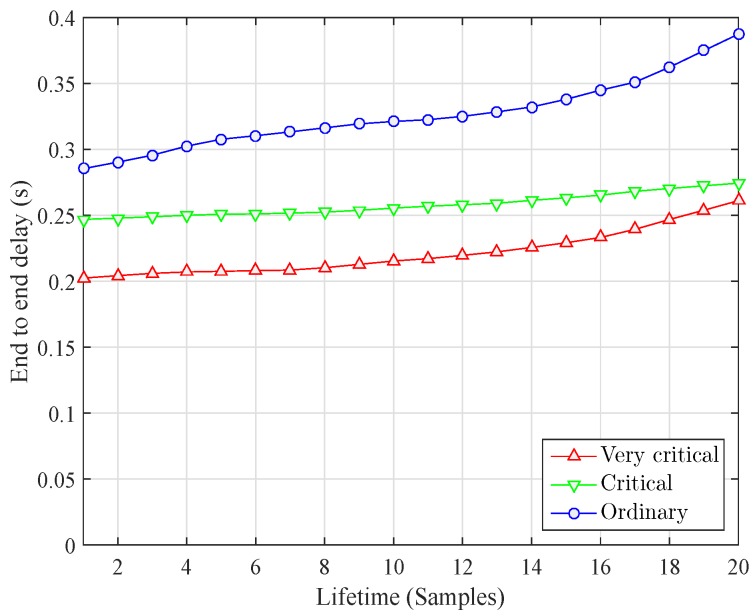
Delay comparison in ESDR.

Average end to end delay of the comparing protocol is calculated using Equation (13) and is plotted in Figure 6. ESDR performs better than its counterparts in terms of average end to end delay. Packet segregation causes significant decrease in delay. ESDR put comparatively lesser forwarding overhead on nodes and hence these nodes cause less retransmission and delay. It is also important to note that delay in DBR is lesser than EEDBR as the later selects forwarders on the basis of energy. Hence a node with lesser depth but more energy can become forwarder involving more number of nodes in forwarding process, which eventually accumulates into larger transmission delays. Delay comparison between different types of nodes in ESDR is given in Figure 7. Delay of normal packets is calculated as the sum of delay from a normal node to nearest courier node and the delay that would have been suffered by an acoustic signal from that courier node to surface sink. Average end to end delay suffered by normal packets is much more than the delay sensitive packets. Very critical packets are forwarded using higher priority values in forwarding functions so they suffer minimum average delay. Critical packet delay remains in between very critical and normal packets. Moreover as the delay in each type increases as the nodes start die out and large distance communication becomes more prominent.

#### 6.1.3. Throughput

**Figure 8:**
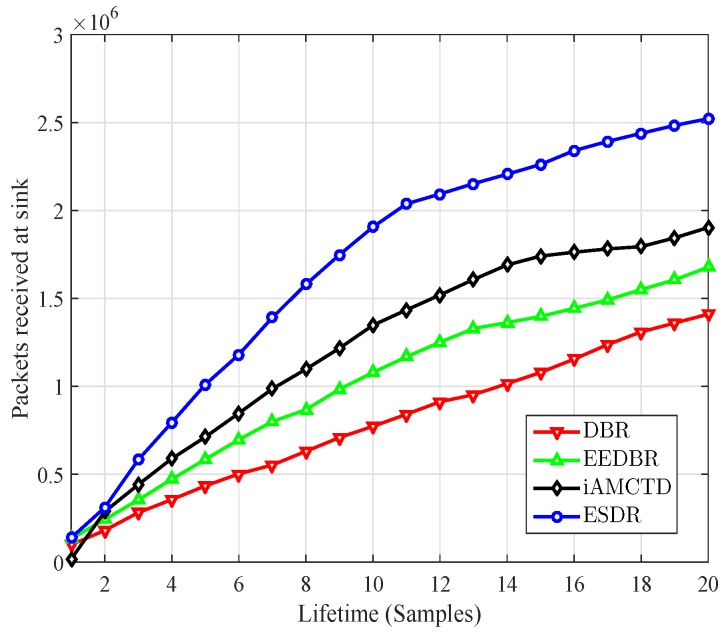
Throughput.

Throughput of the entire network is measured in terms of total number of packets received at BS (Base Station). The comparison for total number of packets successively received at BS is shown in Figure 8. ESDR outperforms its counterparts in terms of throughput by considerable margin. This is because packet loss in ESDR is minimum due to packet segregation strategy. Two third of generated packets are sent to courier nodes available in underwater environment. There is very minute packet loss in this transmission since it requires maximum one hop communication. Only one third of total generated packets are broadcasted to surface sink. In previous protocol holding time value was depending on forwarding function which itself was having inconsistent values. In ESDR Improved forwarding function results into optimum values of holding time which eventually ensures decreased collision and lesser packet loss. So overall comparison, depicted in Figure 8 results into increased throughput of ESDR.

**Figure 9:**
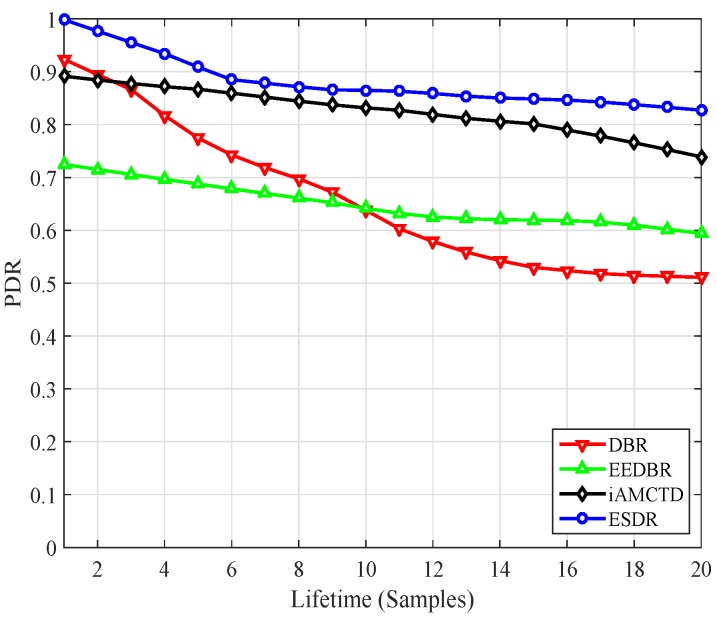
PDR.

Another parameter to get the insight in terms of throughput is Packet Delivery Ratio (PDR) shown in Figure 9. PDR throughout the lifetime is calculated downsampled and plotted for all comparing schemes. Improved forwarding function and efficient adaptive mobility of courier node cause the increase PDR value for proposed protocol. Moreover since approximately 66% of the generated packets are transmitted to courier nodes which may cause delay but ensure reliable delivery of packets destinations resulting with improvement in PDR and throughput as in Figure 9.

#### 6.1.4. Transmission Loss

**Figure 10:**
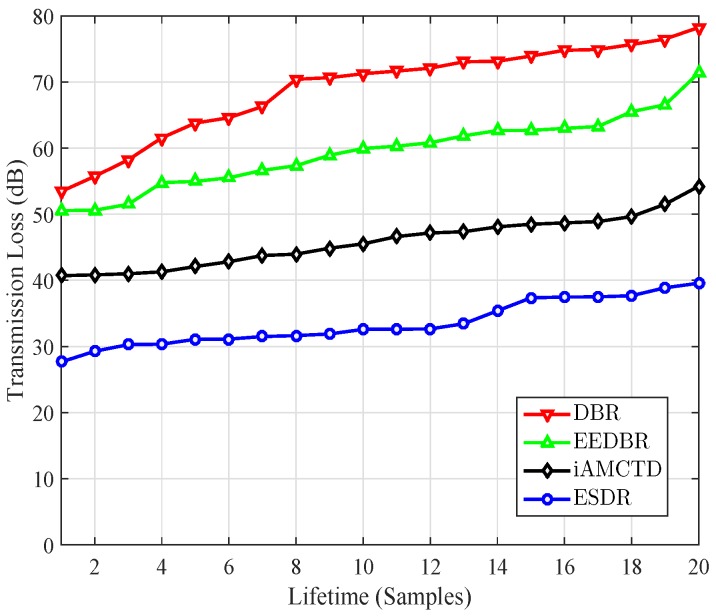
Transmission loss.

Transmission loss is dependent on; number of transmissions that a packet undergo during multi-hop communication, attenuation loss of the signal, and bandwidth efficiency. It is calculated using thorp attenuation formula given in Equation (2). Major contribution to transmission loss is the spreading of the acoustic wave as it propagates away from the source for longer distances in underwater and cause greater transmission losses. Transmission loss for all comparing schemes is calculated and samples from whole lifetime taken in ascending order are plotted in Figure 10. ESDR performs best among all comparing protocol in terms of transmission loss as well. This is due the fact that a major part of data generated is transferred to patrolling sinks and is not suffered to long distances spreading losses. Moreover premature death of intermediate nodes in previous schemes cause increase in transmission loss for their respective protocols.

## 7. Conclusions and Future Work

In this work, we have proposed a novel and energy efficient approach to accommodate delay sensitivity requirements with increased network lifetime for UWSN. We have formulated a strategy in which three types of events with different probability of occurrence are generated and their efficient forwarding is planned. Forwarding functions are analyzed and redefined and holding time calculation process is also improved. However, probability of occurrence for each type of event remains unchanged throughout the lifetime. In the future we intend to implement event segregation approach with varying provability of occurrence of normal, critical and very critical events. In this work, the generated events type (and hence node type) is randomly selected but in future type of event selection is intended to be related with it depth, remaining energy or any other relevant parameter.

## Figures and Tables

**Table 1:** Parameters Ranges w.r.t Threshold.

Properties	Minimum Values			Maximum Value
	$d_{\mathrm{th}}=20$ m	$d_{\mathrm{th}}=40$ m	$d_{\mathrm{th}}=60$ m	
Deptd Difference $\left(l_{i}\right)$	20 m	40 m	60 m	100 m
Absolute Depth $\left(D_{i}\right)$	100 m	100 m	100 m	500 m
Absorption Coefficient (Abc) (km)	0.1990 dB	0.3980 dB	0.5969 dB	0.9949 dB
Scattering Coefficient (Sc) (km)	−25.485 dB	−20.969 dB	−18.328 dB	−15 dB
(Scm)	19.5154 dB	24.0309 dB	26.6723 dB	30 dB
Total Attenuation (At)	−25.286 dB	−20.571 dB	−17.731 dB	−14.005 dB
	19.7144 dB	24.4289 dB	27.2692 dB	30.9949 dB
Attenuation A(l,f)	0.003	0.0088	0.0169	0.0398
	93.6 m${}^{-1}$	277.3 m${}^{-1}$	533.2 m${}^{-1}$	1245.4 m${}^{-1}$
Noise N(f)	20.925 dBreμPa or 1.12 $\times 10^{-5}$ Pa			
	$3.29\times 10^{-8}$	$9.75\times 10^{-8}$	$1.87\times 10^{-7}$	$4.42\times 10^{-7}$
Attenuation Noise Product A(l,f) ×N(f)	$1\times 10^{-3}$	$3.1\times 10^{-3}$	$5.9\times 10^{-3}$	$14.1\times 10^{-3}$

**Table 2:** Forwarding functions ranges.

Protocol	Forwarding Function	Minimum Value	Maximum Values		
			$d_{\mathrm{th}}=20$	$d_{\mathrm{th}}=40$	$d_{\mathrm{th}}=60$
	$W_{1}$	0.0020	70	70	70
AMCTD	$W_{2}$	0.0143	500	500	500
	$W_{3}$	0.0020	70	70	70
iAMCTD	LSNR	$4.52\times 10^{6}$	$6.07\times 10^{7}$	$2.05\times 10^{7}$	$1.06\times 10^{7}$
	SQI	0	$3.03\times 10^{5}$	$1.33\times 10^{5}$	$1.01\times 10^{5}$
	ECF	0	70	70	70
	DDF	0	$3.03\times 10^{4}$	$1.33\times 10^{4}$	$1.01\times 10^{4}$

**Table 3:** Centers of elliptical paths.

*i*	$h_{i}$	$k_{i}$
1	0	140
2	0	380
3	0	620
4	0	860

**Table 4:** Parameters name and value.

Parameter Name	Value
Initial Energy ($E_{o}$)	70 J
Number of nodes |N|	224
Number of courier nodes |C|	4
Maximum Depth ($D_{max}^{\prime }$)	1000 m
Depth Margin 1 ($D_{1}^{\prime }$)	250 m
Depth Margin 2 ($D_{2}^{\prime }$)	750 m
First Death threshold $N_{th_{1}}$	75
Second Death threshold $N_{th_{2}}$	150
Depth threshold 1 ($d_{th_{1}}$)	60 m
Depth threshold 2 ($d_{th_{2}}$)	40 m
Depth threshold 3 ($d_{th_{3}}$)	20 m
Transmission Range ($R_{T}$)	100 m
Speed of acoustic signal (*v*)	1500 ms${}^{-1}$
Priority value for very critical nodes ($p_{v}$)	2
Priority value for critical nodes ($p_{c}$)	1.5
$p_{vc}$	0.111
$p_{cr}$	0.222
$p_{no}$	0.666
